# Unpredictable caregiving is associated with disrupted neurophysiological measures of attention and autonomic function in three-month-old infants

**DOI:** 10.1016/j.dcn.2026.101688

**Published:** 2026-02-04

**Authors:** Denise Werchan, Annie Aitken, Lissete Giménez, Stephen Braren, Natalie H. Brito

**Affiliations:** a University of California, Irvine, Department of Psychology, United States; b New York University, Department of Applied Psychology, United States; c Columbia University, Department of Psychology, United States

**Keywords:** Infants, Attention, EEG, Caregiving

## Abstract

The early caregiving environment exerts a powerful influence over attention and autonomic function in early infancy, which are foundational systems relevant for learning and the development of more complex cognitive and emotional regulation systems. Increasing evidence also implicates the predictability of caregiver sensory signals as a key dimension of the early environment, but few studies have examined impacts on neurophysiological systems relevant to attention and autonomic function in early infancy. The current study examined associations among caregiver sensory predictability, infant autonomic nervous system function (indexed via HRV), and behavioral and neurophysiological measures of sustained attention in a sociodemographically-diverse sample of three-month-old infants (N = 104 infants, 64 males; 51% Hispanic/Latino). The patterning of caregiver sensory signals was assessed by micro-coding for transitions in caregiver visual, auditory, and tactile signals during a semi-structured parent-child interaction and the unpredictability of these sequences was calculated using Shannon’s entropy. Results indicated that infants who were exposed to more unpredictable patterns of caregiver sensory signals demonstrated reductions in relative frontal theta EEG power during periods of sustained attention, a neural marker reflecting poorer information processing and attentional control. Moreover, unpredictable caregiver sensory signals also predicted lower baseline HRV in infants, a pattern indicative of dysregulated ANS function. In turn, infant baseline HRV also predicted altered physiological indices of sustained attention. These findings provide preliminary evidence into the importance of the predictability of caregiver sensory signals in shaping developing cortical circuitry and physiological systems relevant to attentional processing from the first months of postnatal life.

The early rearing environment has a profound impact on brain and behavioral development. Predictable caregiver responses to infant signals are theorized to be a species-expectant experience necessary for healthy cognitive and socioemotional development (Davis and Glynn, 2024, Ugarte and Hastings, 2024). Evidence in 6–12-month-old infants show that unpredictable caregiver sensory and affective signals predict worse long-term cognitive and socioemotional outcomes and increased risk of psychopathology in later childhood (Davis et al., 2017, Davis et al., 2019; Noroña-Zhou et al., 2020). Yet, few studies have examined associations between caregiver predictability and infant attention, a foundational skill that allows infants to filter distractions and select relevant inputs for learning and behavior (Carrasco, 2011). Potential impacts of caregiver sensory inputs on entraining infant attentional systems may be especially salient over the first months of postnatal life, a time in which infant attention develops rapidly and when caregivers serve as a primary source of sensory input (Sullivan et al., 2011). Indeed, the majority of multimodal input that infants experience during early life consists of caregiver-related stimuli, due to constraints on infants’ sensory processing abilities (e.g., poor visual acuity; (Maurer and Lewis, 1999, Turkewitz and Kenny, 1982) combined with the close proximity of infants to their caregivers in early life (Barnett et al., 2022). Yet, prior studies have primarily focused on infants over 6 months of age, and it is unclear whether effects of caregiver sensory predictability on infant neurodevelopment are evident in early postnatal life. We expand upon these gaps in the literature by investigating associations between the predictability of caregiver sensory input and behavioral and neurophysiological measures of attention in three-month-old infants.

Attention is an early emerging cognitive capacity that allows infants to sample information and learn about their environment (Amso and Scerif, 2015). Sustained attention, or the ability to maintain attention over time, can be measured from the first months of postnatal life and continues to develop rapidly across infancy and childhood in support of more complex behavior (Colombo, 2001). Sustained attention is commonly indexed in infancy based on the length of time spent visually attending to a stimulus or task. Physiological changes are also observed when infants are engaged in sustained attention, such that infants’ heart rate decreases relative to pre-stimulus levels (i.e., heart rate decelerations; Reynolds and Richards, 2008; Richards, 2010). These physiological measures may be a more reliable indicator of the quality of infants’ sustained attention, particularly in infants under four months of age who are more likely to experience ‘sticky’ fixation (Atkinson et al., 1992). Indeed, prior work suggests that the amplitude of infants’ heart rate decelerations while visually attending to a stimulus is positively associated with enhanced memory encoding and reduced distractibility (Richards, 2010). Studies using EEG in 10–12-month-olds similarly suggest that increases in frontal theta power during sustained attention relative to baseline levels may be a neural marker of the quality of attentional processing (Xie et al., 2018). These associations also strengthen from 6- to 12-months of age (Xie et al., 2019), and are predictive of longitudinal developmental outcomes. For instance, recent work indicates that increases in frontal theta power during sustained attention at 3-months of age are predictive of visual recognition memory performance at 9-months of age (Brandes-Aitken et al., 2023).

The development of infant attention is thought to be shaped by maturation of the autonomic nervous system (ANS). The ANS is involved in regulating attentional responses to external stimulation, and is commonly measured in infants using heart rate variability (HRV), a measure of the intervals between heartbeats (Acharya et al., 2006). Prior work indicates that variability in infants’ baseline HRV is predictive of individual differences in their attentional processing capacity. For example, higher baseline HRV in 3–6-month-olds has been found to predict larger and more sustained heart rate decelerations during focused attention, as well as less susceptibility to external distractions (Casey and Richards, 1988, Richards, 1985, Richards, 1987, Richards, 1989). Peripheral indices of ANS dysregulation, in the form of lower HRV, are similarly linked with variation in attention and sensory responsivity in children ranging from 7 to 15 years of age with neurodevelopmental disorders (Lory et al., 2020, Bellato et al., 2022). However, while many studies report positive associations between infant autonomic function and attention, findings in this area are not uniformly consistent. For instance, a recent systematic review of 933 infants suggests that these associations are less consistent particularly among older infants or in studies using observational measures of neurodevelopment (Aldrete-Cortez and Tafoya, 2024)

Variability in the patterning of sensory input from caregivers may serve as a critical scaffold of early attention development. For example, during a feeding routine, one caregiver may hold their baby in a steady position and use a calm rhythmic voice while offering each spoonful (“here comes the train! Choo choo”). The predictable timing and sequencing of their movements and vocal cues may help the baby to sustain their attention and remain engaged. However, another caregiver may randomly look at their phone in between spoonfuls, while also varying between talking softly to their baby and shouting to someone across the room. In this example, the inconsistent timing and sequencing of the caregiver’s movements and vocal cues may make it harder for the baby to anticipate what will happen next and focus their attention.

The ostensible importance of predictable sensory inputs for scaffolding attention is supported by animal models, which indicate that the statistical patterning of sensory input helps entrain developing cortical circuits relevant to attention and information processing systems (Chang and Merzenich, 2003, Dorrn et al., 2010, Espinosa and Stryker, 2012a, Khazipov et al., 2004). Similarly, behavioral studies in human infants suggest that 7–8-month-olds prefer to attend to patterns of intermediate complexity and predictability (Kidd et al., 2012, Kidd et al., 2014). Other convergent evidence comes from work showing that 6–10-month-old infants rapidly extract predictable patterns from sequences of multimodal inputs, which subsequently guide attention in novel contexts (Tummeltshammer and Amso, 2018, Werchan and Amso, 2020). More broadly, naturalistic variations in sensory input within the proximal environment have also been linked to individual differences in infant attention development from early in infancy. For instance, three-month-old infants residing in homes with more unpredictable patterns of ambient noise exposure show poorer behavioral and neurophysiological measures of attention (Werchan et al., 2022). Similarly, 12-month-old infants who are exposed to higher and more rapidly fluctuating environmental noise also show reductions in visual sustained attention (Wass et al., 2019). However, these studies have focused on broader patterns of ambient noise exposure measured using daylong recordings, rather than the micropatterns of caregiver sensory input (e.g., speech, touch) that unfold in the context of parent-child interactions.

It is also possible that caregiver sensory signals may also influence infant ANS function, with downstream consequences on infant attention and neurocognitive development. This possibility is supported by recent evidence suggesting that the infant ANS is highly responsive to fluctuations in the early environment and caregiving behaviors. For example, 12-month-infants who experience greater variability in environmental noise show more unstable autonomic arousal patterns (Wass et al., 2019). Other work has found that maternal sensitivity is positively associated with baseline levels of HRV at 6 and 12 months (Bosquet Enlow et al., 2014, Köhler-Dauner et al., 2022). Three-month-old infants of mothers reporting elevated psychosocial stress have also been found to have lower baseline HRV (Brandes-Aitken et al., 2024), which could reflect negative impacts of maternal stress or anxiety on the predictability of caregiving (Holmberg et al., 2020).

Collectively, these findings suggest that variability in caregiver sensory signals may shape trajectories of neurocognitive development, in part through impacts on infant attentional processing and ANS function in early postnatal life. However, potential impacts of caregiver sensory predictability on infant ANS function and neurophysiological measures of attention have not been investigated in young infants. As such, the goals of the current study are twofold. First, we evaluate the impacts of caregiver sensory predictability during a parent-child-interaction on behavioral (looking time), physiological (heart rate deceleration), and neural (frontal theta) measures of sustained attention in three-month-old infants. We then examine whether caregiver sensory predictability is associated with variability in infant ANS function, indexed via baseline HRV. In doing so, we aim to increase understanding of the potential underlying pathways through which variations in the predictability of early caregiving behaviors may be translated into divergent long-term neurodevelopmental outcomes.

## 1 Method

### 1.1 Participants

**Fig. 1:**
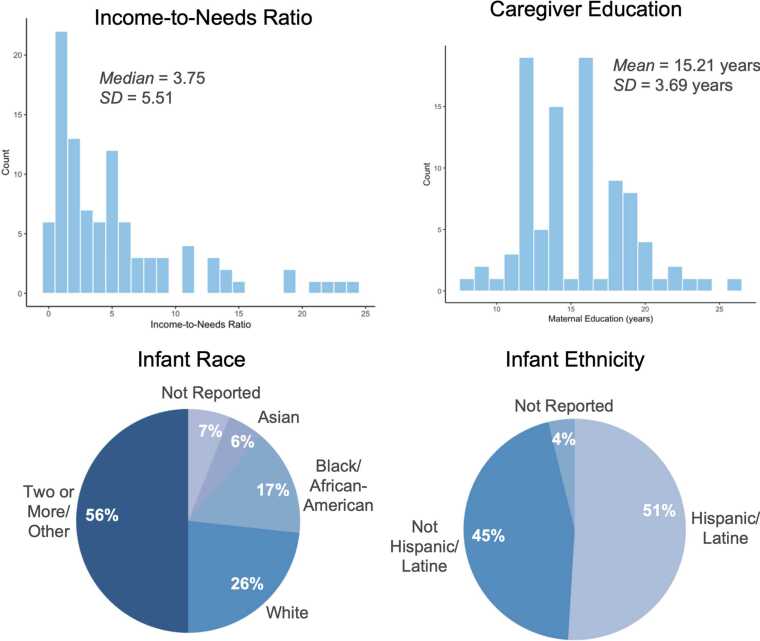
Sample sociodemographic characteristics. Note that caregiver education reflects educational attainment for the caregiver who completed the parent-child interaction in the study (95 % mothers, 5 % fathers).

The analytic sample consisted of 104 three-month-old infants drawn from a larger longitudinal study (64 males, *M* age = 3.47 months, *SD* =.39 months; *range* = 2.20 4.37; for related publications describing the sample, see Brito et al., 2022; Werchan et al., 2022; Brandes-Aitken et al., 2023, Brandes-Aitken et al., 2024). Families were recruited (05/2018–12/2019) from community events, family services, health care providers, and flyers posted at local businesses around New York City and were tested at a university laboratory located in lower Manhattan. Participants were excluded from enrollment in the study on the basis of birth before 36 weeks’ gestation or presence of developmental disorders. Participant sociodemographics are reported in Fig. 1. Families were compensated for their time and transportation was provided to all families. All procedures were approved by the New York University IRB.

Sensitivity analyses were used to determine minimally detectable effect sizes, as we had a fixed sample drawn from a larger longitudinal study. Given a type I error rate of.05 and power > =.80, sensitivity analyses indicate that our sample size is adequately powered to detect relatively small effects (*ß* > 0.25). As such, our sample size is sufficient to detect expected medium effect sizes, estimated from prior research (e.g., Werchan et al., 2022).

### 1.2 Procedure

Families participated in (1) a resting infant ECG recording to measure baseline ANS function, (2) a semi-structured parent-child interaction task to measure caregiver sensory predictability, and (3) a sustained attention task to measure infants’ behavioral, neural, and autonomic responses to attention-eliciting stimuli. Families also completed demographic questionnaires to obtain information on infant age, race and ethnicity, educational attainment, and annual household income. Family income-to-needs ratio (ITN) was calculated from the total household income divided by the federal poverty line for the corresponding number of adults and children in the home.

### 1.3 Measures

**Baseline HRV.** Infant baseline heart-rate data were collected using a Physio16 (EGI) ECG device while infants watched engaging, non-social stimuli (e.g., bubbles, spinning wheel) while seated on their caregivers’ laps in a dimly-lit room. Infant electrodes were placed on the upper chest on the pectoralis muscles 3–4 in. apart. The intended duration of the baseline recording was 10 min. Variability in the actual length of this period reflects instances of infant fussiness or reduced tolerability, which occasionally required pauses or early termination of the recording. Infants provided an average of 274 s of useable baseline ECG data in the current sample (min = 95 s, max = 312 s). Following prior studies (Brandes-Aitken et al., 2024), raw ECG files were edited and processed using the QRSTool software to automatically detect R-R intervals and remove artifacts. All processed recordings were visually inspected to identify detection errors, including missed beats, spurious detections, and artifacts. Corrections were made manually using QRSTool’s point-and-click editor: missed beats were added, false detections were removed, and ectopic or noisy intervals were replaced with interpolated values based on adjacent valid R-R intervals.

The cleaned ECG data were then linear interpolated to a uniform sampling grid (100 Hz) before computing HRV metrics. The root mean square of successive differences (RMSSD) was used to index infant baseline HRV. The RMSSD reflects the beat-to-beat variance in heart rate and is a primary time-domain measure used to estimate vagally-mediated baseline autonomic function (Shaffer and Ginsberg, 2017). To account for the known relationship between heart rate and HRV (Laborde et al., 2017, Sacha, 2014), mean heart rate during the baseline recording was included as a covariate in all analyses involving RMSSD. In addition, we winsorized baseline HRV at the 5th and 95th percentiles to account for outliers in baseline HRV scores.

**Caregiver sensory predictability.** Each caregiver-infant dyad was video-recorded during a semi-structured 5-min caregiver-infant play interaction. Caregivers were given a standardized set of toys including a book, stacking cups, and a rattling ball, and they were instructed to play like they normally would at home. Established coding schemes (Davis et al., 2017) for evaluating caregiver auditory, visual, or tactile sensory signals to the infant were coded using BORIS – an open-source event-logging software for audio/visual coding (Friard and Gamba, 2016). Auditory signals included all caregiver vocalizations (e.g., talking, laughing). Visual signals included caregiver point or manipulation of a toy or object while the infant was looking at it. Tactile signals involved all instances of physical contact (e.g., holding, touching) that were initiated by the caregiver. Coders were blind to all information on study participants. Interrater reliability was calculated for 20 % of the videos. Raw agreement was 93 % and Cohen’s κ averaged.69, indicating substantial interrater reliability.

The conditional probabilities of transitioning between all possible combinations of auditory, visual, and tactile sensory signals were calculated from the coded parent-child interactions. The predictability of caregiver sensory signals was estimated by the entropy rate of the transition series, with higher entropy values reflecting less-predictability. Further details and scripts for calculating entropy rate are provided in Davis et al. (2017) and available at https://contecenter.uci.edu/shared-resources/. The vast majority of caregivers completing the parent-child interaction were mothers (95 %), but fathers consisted of a small subset of caregivers (5 %). We verified that there were no differences in caregiver entropy rates between mothers (*M* = 0.78, *SD* = 0.32) and fathers (*M* = 0.97, *SD* =.27), *t*(84) = 1.34, *p* = .19.

**Sustained attention.** A sustained visual attention task adapted from Xie et al. (2018) was administered to measure infants’ behavioral, neural, and autonomic responses to attention-eliciting stimuli. Infants sat on their caregivers’ lap while they watched a dynamic 281-second video on a large computer monitor in a dimly lit room. The video consisted of several characters from *Sesame Street* that moved from side to side, disappeared, sang, and danced. These videos have been demonstrated to elicit periods of sustained attention in young infants (Xie et al., 2018). A camera under the monitor recorded the infants’ faces, while a camera behind the participants recorded the stimuli. Visual attention to the stimuli were manually coded from the recorded videos using the Net Station 5.1 software.

As in prior studies, ECG measures of heart-rate deceleration were collected during the task to identify periods of heightened arousal (Courage et al., 2006, Mallin and Richards, 2012, Reynolds and Richards, 2008, Richards, 2010). Infant ECG data were edited and processed using the QRSTool software to remove artifacts and identify heartbeats, and the inter-beat interval (IBI), defined as the latency period between the R waves of two heartbeats, was then extracted (see baseline HRV section for more details on ECG processing). Following the methods of Xie et al. (2018), phases of attention were identified based on infant looking behavior and measures of heart rate deceleration. Specifically, sustained attention phases began when the infant was looking at the screen *and* five consecutive IBI values were higher than the median of the five baseline IBI values preceding the sustained attention phase. Sustained attention phases ended when five consecutive IBI values were lower than the median of the preceding five. Baseline values were reset each time sustained attention was terminated.

A behavioral index of sustained attention was quantified by the total duration of time spent in periods of heart rate defined sustained attention. In addition, infants’ average heart rate deceleration during periods of sustained attention was used to index infants’ autonomic arousal in response to attention-eliciting stimuli. Heart rate deceleration was quantified as the change in infants’ average IBI during phases of sustained attention relative to average IBI during the 5 s baseline interval immediately preceding the onset of the first stimulus, with more positive scores indicating greater decelerations. This ensured that IBI scores reflected relative change from each infants’ baseline rather than absolute IBI values.

Frontal EEG data were acquired during the sustained attention task using a 64-channel HydroCel Geodesic Sensory Net (Electrical Geodesic, Inc., Eugene, OR) and amplifier (Electrical Geodesic, Inc., Eugene, OR; EB NEURO S.p.A., Firenze, Italy). Electrode impedances were kept below 100 KΩ and the sampling rate was recorded at 1000 Hz. Relative oscillatory power in the Theta (4–6 Hz) frequency range was averaged across all channels in the frontal region (electrode #: 2, 3, 5, 6, 9, 10, 11, 12, 13, 14, 57, 59, 60) during heart-rate defined phases of sustained attention and inattention. Change scores of relative frontal theta power during periods of attention relative to inattention were used to index the neural correlates of sustained attention.

### 1.4 EEG data processing

All EEG files were processed in batch electroencephalography automated processing platform (BEAPP) software to ensure standardization in data processing and cleaning across all files (Levin et al., 2018). Data preprocessing was carried out using the Harvard Automated Processing Pipeline for EEG (HAPPE; Gabard-Durnam et al., 2018). First, a 1 Hz high-pass and 100 Hz low-pass filter was applied to each EEG dataset. Second, the data was resampled with interpolation to 250 Hz, following guidelines for further HAPPE processing. The third step involved artifact removal and included CleanLine’s multitaper approach to removing 60 Hz electrical noise, bad channel rejection, and wavelet-enhanced ICA for artifact rejection with automated component rejection through the Multiple Artifact Rejection Algorithm (Winkler et al., 2011) in EEGLAB. Bad channels that were initially rejected were repopulated using spherical interpolation to reduce bias in re-referencing and the signal was mean detrended. Finally, each EEG file was segmented into 1-second windows and each segment was assessed for remaining artifacts. Segment rejection thresholds were determined according to HAPPE’s automated rejection criteria (Gabard-Durnam et al., 2018), which uses amplitude thresholding and assessment of segment likelihood using joint probability calculations. During these data cleaning procedures, the average percentage of independent components that were rejected was 44.0 %, the average artifact probability for retained components after ICA was 18.6 %, and the average percentage of segments that were rejected was 1.13 %. These data loss metrics are in line with those reported in prior infant and adult EEG studies (Gabard-Durnam et al., 2018). EEG power decomposition was then accomplished using Matlab’s fast fourier transformation using hanning windowing to decompose into power for 1-second segments for each channel. Summed power within each frequency band was averaged across all segments and normalized by a log base 10 transformation. Segments exceeding 3 SD + /- micro-volts from the median were excluded from analysis (Xie et at., 2018), resulting in an average removal of 5 segments (out of a total of 250 segments on average), with a minimum of 1 and maximum of 6 across all infants. Infants with less than 20 usable segments were excluded from analysis (N = 3).

### 1.5 Statistical analyses

Possible sociodemographic (i.e., race/ethnicity, caregiver age, primary caregiver educational attainment, family income-to-needs ratio) and infant (i.e., age at test, biological sex at birth) variables that might influence predictors or outcomes were evaluated using correlations, chi square tests, and *t* tests (see Supplemental Table 1 for results). These candidate covariates were selected based on prior literature suggesting these as potentially relevant factors in infant attention, ANS, or EEG research (Adams et al., 2024, Brito et al., 2016, Christensen et al., 2023, Clarke et al., 2001, Sandre et al., 2025). Variables showing significant associations with caregiver entropy rate or infant behavioral, physiological, or neural outcomes at the level of *p* < 0.10 were flagged for inclusion as covariates in all analyses. Infant sex and infant age at test met this criterion and were thus included as covariates in all analyses. We additionally controlled for infant gestational age at birth in our sample (*M* = 39.21 weeks, *SD* = 1.22 weeks, *range* = 36 – 42 weeks), given that gestational age has a significant and long-lasting impact on the EEG power spectrum even amongst non-preterm infants (e.g., Luotonen et al., 2025) and is thus considered a standard covariate in infant EEG research.

Missing data on the study variables ranged from 11 % to 23 %, and Little’s MCAR test indicated that data were missing at random (p = .69; see Supplemental Figure 1 for proportions and patterns of missing data for all study variables). Full information maximum likelihood (FIML) was used to account for missing data in all analyses, as FIML produces unbiased parameter estimates when data are missing at random.

Multiple linear regressions with maximum likelihood estimation were used to evaluate associations between caregiver sensory predictability, measures of infant sustained attention, and infant baseline autonomic function. All analyses were run in R using the *lavaan* package. As additional sensitivity checks, all analyses were repeated using (a) maximum likelihood estimation with robust standard errors, (b) multiple imputation with chained equations (using the MICE package in R, with 25 imputations), and (c) listwise deletion. Results were consistent across all robustness checks.

## 2 Results

**Fig. 2:**
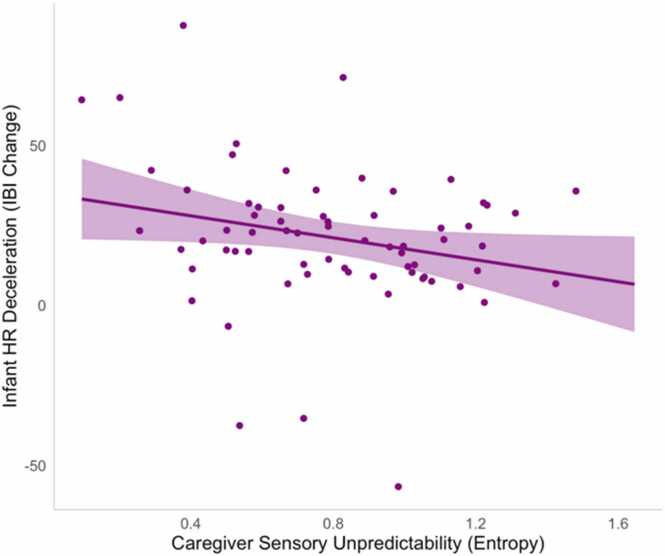
Unpredictable caregiver sensory signals were associated with reduced heart rate decelerations during sustained attention (note: higher IBI values reflect greater decelerations).

**Fig. 3:**
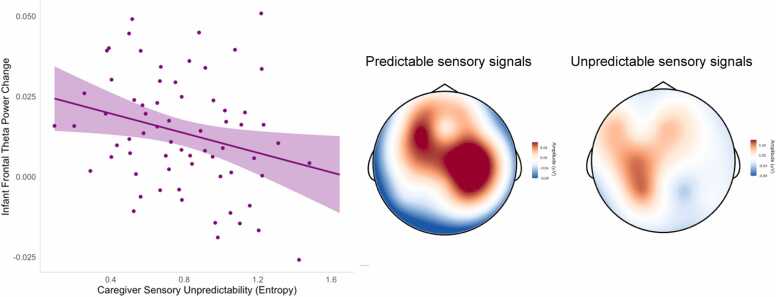
Unpredictable caregiver sensory signals were associated with reduced frontal theta power during sustained attention. For illustration purposes, topo plots are shown for the 10 infants with the highest and lowest caregiver sensory unpredictability scores (entropy rate).

Descriptive statistics for all study variables are presented in Table 1. We first examined infants’ behavioral, neural, and autonomic responses to attention-eliciting stimuli during the sustained attention task using multiple linear regressions (full results reported in SI Table 2). All analyses controlled for infant age at test, infant sex, and gestational age at birth. Results indicated that unpredictable caregiver sensory signals were not associated with the total duration of infants’ sustained attention, *ß* = -.07, *p* = .565. However, there was a significant negative effect of unpredictable caregiver sensory signals on the magnitude of infants’ heart rate decelerations during sustained attention, *ß* = -.26, *p* = .040 (Fig. 2). Similarly, we also found that higher unpredictability was associated with reduced frontal theta power during periods of sustained attention relative to inattention, *ß* = -.24, *p* = .041 (Fig. 3). These negative associations suggest that more unpredictable caregiver sensory signals are correlated with lower autonomic arousal and reduced frontal EEG power in response to attention-eliciting stimuli.

**Table 1:** Descriptive statistics.

	**Mean**	**SD**	**Range**	**Skew**	**Kurtosis**
Caregiver Entropy Rate	0.79	0.32	[0.09–1.65]	0.23	-0.61
Frontal Theta Δ	0.01	0.02	[-0.03–0.05]	0.15	-0.50
HR Deceleration (Δ IBI)	20.16	22.83	[-56.58–87.38]	-0.39	2.19
Sustained Attention Duration (s)	124.13	67.12	[10 – 276]	0.37	-0.71
Baseline HRV	11.23	4.65	[4.86–27.85]	1.18	0.59

**Fig. 4:**
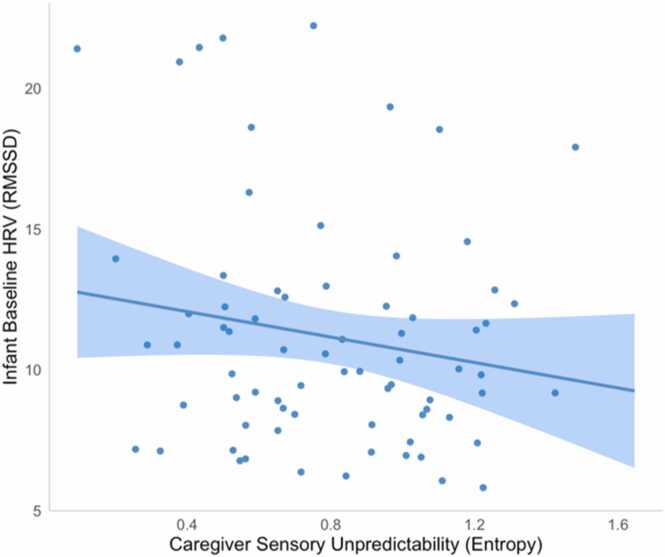
Unpredictable caregiver sensory signals were associated with lower infant baseline HRV.

Multiple linear regression was next used to test whether unpredictable caregiver sensory signals were associated with infant baseline HRV, indexed via RMSSD at rest. We controlled for baseline heart rate, infant age, infant sex, and infant gestational age at birth. Results indicated that more unpredictable caregiver sensory signals were associated with lower levels of infant baseline HRV, *ß* = -.25, *p* = .026 (Fig. 4; full results reported in SI Table 3). Thus, this finding suggest that unpredictable caregiver sensory signals may have negative effects on infants’ baseline autonomic function, indexed via lower HRV at rest.

**Fig. 5:**
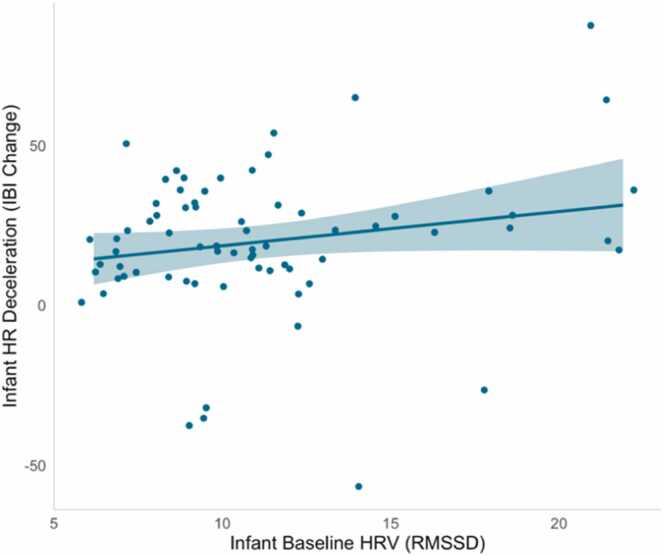
Lower infant baseline HRV predicted reduced heart rate decelerations during sustained attention.

Finally, we evaluated associations between infants’ baseline HRV and their subsequent behavioral, neural, and autonomic responses to attention-eliciting stimuli. We again controlled for infant age, infant sex, and infant gestational age at birth. Multiple linear regressions indicated that lower baseline HRV were not associated with the duration of sustained attention, *ß* = -.02, *p* = .892, nor with infants’ frontal theta power during sustained attention, *ß* = .08, *p* = .496. In contrast, however, we observed that lower infant baseline HRV was associated with smaller heart rate decelerations during sustained attention, *ß* = .25, *p* = .047 (Fig. 5; full results reported in SI Table 4). As a robustness check to assess potential nonlinearity, we included a quadratic term for HRV in the model. The quadratic term was not significant (*p* = .541), indicating that the relationship between HRV and attention is adequately captured by a linear association and is not disproportionately influenced by extreme baseline HRV values.

In a final exploratory analysis, we examined potential sex effects, which indicated no significant or trending sex differences in associations between caregiver entropy rates and infant outcomes (SI Table 5).

## 3 Discussion

The predictability of the early environment – and caregiving behaviors in particular – is shown to exert powerful effects on long-term cognitive and socioemotional outcomes and risk for psychopathology in later childhood (Davis et al., 2017, Davis et al., 2019, Noroña-Zhou et al., 2020). However, the majority of these studies have examined the predictability of caregiving behaviors in infants over 6 months of age, and few studies have examined impacts of caregiver sensory signals on shaping neurocognitive systems critical for learning and information processing in the first months of infancy. As such, the current study examined associations between unpredictable caregiver sensory signals, infant ANS function (indexed via baseline HRV), and behavioral, neural, and autonomic responses to attention-eliciting stimuli in three-month-old infants.

Our results indicated that unpredictable caregiver sensory signals were associated with reduced frontal theta power during sustained attention – a pattern thought to reflect less efficient attention processing (Tan et al., 2024). This finding is consistent with prior work indicating that 3-month-old infants residing in homes with less predictable patterns of ambient noise exposure similarly show attenuated increases in frontal theta power during periods of sustained attention relative to inattention (Werchan et al., 2022). This suggests that similar effects are observed whether sensory predictability is measured through daylong recordings of ambient noise exposure, as in Werchan and colleagues (2022), or through micro-coding of caregiver sensory input during a parent-child interaction, as in the current report. It is possible that our findings may reflect entrainment of developing cortical circuitry to predictable oscillations in sensory input experienced in the external environment (Wass et al., 2022). Indeed, exposure to structured patterns of sensory inputs shapes the development of brain synapses and circuits across auditory, visual, and somatosensory cortical regions (Dorrn et al., 2010, Espinosa and Stryker, 2012b, Rose et al., 2004). Over time, this may have cascading effects on the development of cortical networks relevant to more complex cognitive, behavioral, and emotional processing abilities.

Mirroring these EEG findings, we also found that more unpredictable caregiver sensory signals was associated with reduced autonomic arousal to attention-eliciting stimuli, indicated by smaller heart rate decelerations during sustained attention relative to pre-stimulus levels. In contrast, we observed no effect of caregiver predictability on the total duration of time that infants spent in heart-rate defined periods of sustained attention. It is possible that this null association may reflect the use of compensatory strategies that allow infants to maintain similar behavioral performance despite reduced neurophysiological efficiency. However, in contrast to using duration-based measures of attention alone, measures of infants’ neural and physiological responses to attention-eliciting stimuli are thought to be a stronger indicator of the *quality* of attentional processing (Richards, 2010). For instance, findings in 3–6-month-old infants show that the amplitude of heart rate decelerations during attentional encoding is concurrently associated with less distractibility and better memory for the attended stimuli (Casey and Richards, 1988; Richards, 1997; see also Reynolds, 2015, for review). Similar findings show that the change in infants’ frontal theta power during sustained attention at 3 months of age prospectively predicts better subsequent visual recognition memory at 9 months (Brandes-Aitken et al., 2023). Thus, even if caregiver sensory unpredictability is not associated with behavioral differences in the duration of sustained attention, it may impact the depth of attentional processing, with cascading effects on subsequent learning and memory outcomes.

A second primary finding was that unpredictable caregiver sensory signals were associated with lower baseline HRV in infants. Past research proposes that HRV is indicative of the capacity for top-down cognitive control and adaptive regulation of autonomic responses to external stimulation across the lifespan (Holzman and Bridgett, 2017, Thayer et al., 2009). Specifically, higher baseline HRV is thought to reflect greater flexibility in adapting to changing environmental demands, whereas lower HRV is linked with greater difficulty in sustaining attention over time. Thus, infants who experience more unpredictable sensory signals from their caregivers may demonstrate less capacity to adapt to changing attentional demands and maintain focus. In line with this hypothesis, we found that infants’ who had lower baseline HRV also tended to show reduced frontal theta power and smaller heart rate decelerations during the subsequent sustained attention task. These findings align with prior work in infants showing that infant baseline HRV is a strong predictor of behavioral and physiological indices of focused attention (Richards, 1985, Richards, 1987, Richards, 1989).

Taken together, our results suggest that unpredictable caregiver sensory signals may be associated with altered development of infant attention and ANS function. This finding is consistent with prior work showing that unpredictable noise exposure in the home is associated with unstable patterns of autonomic arousal (Wass et al., 2019). Similarly, other findings in adults demonstrate that individuals who were raised in homes with greater unpredictability exhibit more ‘bottom-up’ or reactive attentional styles – patterns that are consistent with dysregulated ANS function (Wang et al., 2024, Xu et al., 2023). Our findings also align with previous work showing that unpredictable maternal sensory signals are associated with a blunted cortisol response to a stressor (Noroña-Zhou et al., 2020). We extend upon these findings by showing associations between caregiver sensory predictability and altered infant stress physiology, in the form of reduced baseline HRV. However, our findings should also be interpreted cautiously, given that we had a modest sample size and tested infants at a relatively early age – a time in which there is substantial variability in both infant ANS development and attentional processing. Future longitudinal work in larger samples is needed to replicate and extend these findings to better determine the robustness and reliability of these effects.

Together, these findings support the possibility that chronic exposure to unpredictable sensory signals in early life may lead to dysregulated attention and ANS function, with potential downstream effects on the development of more complex cognitive and emotional regulation systems. However, it is important to avoid reducing predictability into a false dichotomy between “good” and “bad”. In everyday contexts, some degree of variability in caregiver sensory input is both inevitable and likely highly adaptive. Fluctuations in a caregiver’s voice, touch, or movement may reflect the natural dynamics of the environment, exposing infants to a range of sensory experiences that allow them to flexibly adapt to the demands present in their unique ecological niche (Werchan and Amso, 2017).

In sum, our findings provide preliminary evidence that greater unpredictability in caregiver sensory signals was associated with reduced frontal theta power and smaller heart rate decelerations during sustained attention, neurophysiological patterns that are indicative of less efficient attentional processing. Moreover, we also observed that unpredictable caregiver sensory signals were negatively associated with infants’ baseline HRV, potentially reflecting dysregulated ANS function and stress physiology. This work increases our understanding of the candidate pathways through which early caregiver sensory signals may shape foundational physiological and neural systems relevant for developing cognitive and emotional regulation systems from the first months of postnatal life.

## Diversity in citation practices

The authors of this paper report its proportions of citations by gender category to be: 30.4 % M/M, 4.3 % M/W, 28.3 % W/M, and 37.0 % W/W.

## CRediT authorship contribution statement

**Stephen Braren:** Investigation, Data curation. **Natalie H. Brito:** Writing – review & editing, Methodology, Investigation, Funding acquisition, Data curation, Conceptualization. **Denise Werchan:** Writing – review & editing, Writing – original draft, Visualization, Formal analysis, Data curation, Conceptualization. **Annie Aitken:** Writing – review & editing, Investigation, Data curation, Conceptualization. **Lissete Giménez:** Validation, Data curation.

## Funding

This work was funded by 10.13039/100000002NIH R00HD086255 to NHB.

## Declaration of Competing Interest

The authors declare that they have no known competing financial interests or personal relationships that could have appeared to influence the work reported in this paper.

## Data Availability

The data and analysis scripts that support the findings of this study are available on request from the corresponding author.
