# Supplementary material for: Unpredictable caregiving is associated with disrupted neurophysiological measures of attention and autonomic function in three-month-old infants
Source: Dev Cogn Neurosci. 2026 Feb 4;79:101688. doi: 10.1016/j.dcn.2026.101688 (PMC12914455; doi:10.1016/j.dcn.2026.101688)
Supplement: Supplementary file 1 — Supplementary material [file mmc1.docx]

**Supplemental Figure 1.** Patterns of missing data for all study variables.

**Supplemental Table 1.** Statistical test results evaluating potential covariates for inclusion.

|  | **Entropy**  **Rate** | **Frontal Theta Δ** | | **HR Deceleration** | | **Attention Duration** | | **Baseline**  **HRV** | |
| --- | --- | --- | --- | --- | --- | --- | --- | --- | --- |
| Infant Race | χ^2^ = 246, *p* = .44 | | χ^2^ = 213, *p* = .43 | | χ^2^ = 219, *p* = .43 | | χ^2^ = 196, *p* = .35 | | χ^2^ = 218, *p* = .50 |
| Infant Ethnicity | *t* = -.01, *p* = .99 | | *t* = -.59, *p* = .55 | | *t* = -.66, *p* = .51 | | *t* = -.05, *p* = .96 | | *t* = 1.10, *p* = .27 |
| Primary Caregiver Age | *r* = .06, *p* = .61 | | *r* = -.16, *p* = .16 | | *r* = -.12, *p* = .30 | | *r* = -.09, *p* = .45 | | *r* = -.06, *p* = .59 |
| Primary Caregiver Education | *r* = .02, *p* = .87 | | *r* = -.03, *p* = .81 | | *r* = -.02, *p* = .88 | | *r* = .09, *p* = .44 | | *r* = .02, *p* = .84 |
| Household Income-to-Needs | *r* = .13, *p* = .24 | | *r* = .01, *p* = .93 | | *r* = .02, *p* = .88 | | *r* = -.01, *p* = .95 | | *r* = .10, *p* = .38 |
| Infant Age at Test | *r* = .11, *p* = .34 | | *r* = -.16, *p* = .18 | | *r* = -.07, *p* = .56 | | *r* = -.20, *p* = .09 | | *r* = -.07, *p* = .51 |
| Infant Sex at Birth | *t* = .84, *p* = .41 | | *t* = -2.38, *p* = .02 | | *t* = -.96, *p* = .34 | | *t* = -1.04, *p* = .30 | | *t* = 1.21, *p* = .23 |

**SI Table 2.** Full regression results examining caregiver sensory signals as a predictor of infant attention.

| **Model** | **Frontal Theta Δ** | | | | | | **HR Deceleration** | | | | | | | | **Attention Duration** | | | |  |  |
| --- | --- | --- | --- | --- | --- | --- | --- | --- | --- | --- | --- | --- | --- | --- | --- | --- | --- | --- | --- | --- |
| *Predictors* | *β* | | | | *p* | | | *β* | | | | *p* | | | | *β* | | *p* | |  |
| Caregiver Entropy Rate | -.24 | | | .041 | | | -.26 | | | | .040 | | | | -.07 | | .565 | |  | |
| Infant Gestational Age | -.12 | | | .293 | | | .02 | | | | .849 | | | | -.10 | | .366 | |  | |
| Infant Age at Test | -.14 | | | .228 | | | -.02 | | | | .843 | | | | -.17 | | .154 | |  | |
| Infant Sex at Birth | .24 | | | .015 | | | .07 | | | | .514 | | | | .09 | | .447 | |  | |
|  |  |  |  | | |  | | |  |  | | |  |  |  |  |  |  |  |  |
|  |  |  |  | | |  | | |  |  | | |  |  |  |  |  |  |  |  |

| **SI Table 3.** Full regression results examining caregiver sensory signals as a predictor of infant baseline HRV. | | | |
| --- | --- | --- | --- |
|  | **Baseline HRV** | | |
| *Predictors* | *β* | *p* |  |
| Caregiver Entropy Rate | -.25 | .040 | |
| Infant Gestational Age | -.06 | .489 | |
| Infant Age at Test | -.07 | .395 | |
| Infant Sex at Birth | -.16 | .123 | |
| Resting Heart Rate | -.37 | .001 | |

**SI Table 4.** Full regression results examining infant baseline HRV as a predictor of infant attention.

| **Model** | **Frontal Theta Δ** | | | **HR Deceleration** | | | | **Attention Duration** | | | |  |  |
| --- | --- | --- | --- | --- | --- | --- | --- | --- | --- | --- | --- | --- | --- |
| *Predictors* | *β* | | *p* | | *β* | | *p* | | *β* | | *p* | |  |
| Infant Baseline HRV | .08 | .513 | | .27 | | .039 | | -.02 | | .905 | |  | |
| Infant Resting HR | .02 | .882 | | .05 | | .714 | | .06 | | .618 | |  | |
| Infant Gestational Age | -.10 | .338 | | .05 | | .651 | | .104 | | .359 | |  | |
| Infant Age at Test | -.18 | .114 | | -.05 | | .656 | | -.18 | | .123 | |  | |
| Infant Sex at Birth | .30 | .008 | | .13 | | .252 | | .09 | | .451 | |  | |

**SI Table 5.** Exploratory analyses examining interactions between caregiver sensory predictability and infant sex on infant outcomes.

| **Model** | **Frontal Theta Δ** | | | | **HR Deceleration** | | | | | | **Attention Duration** | | | | | | | **Baseline HRV** | | | | |  |  |
| --- | --- | --- | --- | --- | --- | --- | --- | --- | --- | --- | --- | --- | --- | --- | --- | --- | --- | --- | --- | --- | --- | --- | --- | --- |
| *Predictors* | *β* | | | *p* | | *β* | | | *p* | | | *β* | | | *p* | | *β* | | | | *p* | | | |
| Caregiver Entropy Rate | -.24 | | .034 | | -.25 | | | .060 | | | -.09 | | | .454 | | | | | -.22 | | | .050 |  |  |
| Infant Sex at Birth | .25 | | .022 | | .65 | | | .651 | | | .07 | | | .544 | | | | | -.15 | | | .173 |  |  |
| Sex X Entropy | .01 | | .965 | | .02 | | | .880 | | | -.12 | | | .330 | | | | | -.01 | | | .960 |  |  |
| Infant Age at Test | -.11 | | .394 | | -.03 | | | .783 | | | -.18 | | | .138 | | | | | -.08 | | | .463 |  |  |
| Infant Gestational Age | -.11 | | .317 | | .01 | | | .873 | | | -.06 | | | .624 | | | | | -.09 | | | .404 |  |  |
| Infant Resting HR | - | | - | | - | | | - | | | - | | | - | | | | | -.35 | | | .001 |  |  |
|  |  |  | | |  | |  | | |  | | |  | | |  | | | |  | | | |  |
|  |  |  | | |  | |  | | |  | | |  | | |  | | | |  | | | |  |
|  |  |  | | |  | |  | | |  | | |  | | |  | | | |  | | | |  |
